# Sustainable High Quality Recycling of Aggregates from Waste-to-Energy, Treated in a Wet Bottom Ash Processing Installation, for Use in Concrete Products

**DOI:** 10.3390/ma9010009

**Published:** 2015-12-25

**Authors:** Philip Van den Heede, Niels Ringoot, Arno Beirnaert, Andres Van Brecht, Erwin Van den Brande, Geert De Schutter, Nele De Belie

**Affiliations:** 1 Magnel Laboratory for Concrete Research, Ghent University, Technologiepark Zwijnaarde 904, Ghent B-9052, Belgium; philip.vandenheede@ugent.be (P.V.H.); nielsringoot10@hotmail.com (N.R.); arno_beirnaert@hotmail.com (A.B.); geert.deschutter@ugent.be (G.D.S.); 2 Indaver nv, Dijle 17a, Mechelen B-2800, Belgium; andres.van.brecht@indaver.be (A.V.B.); erwin.van.den.brande@indaver.be (E.V.B.)

**Keywords:** municipal solid waste incineration, bottom ash, concrete, aggregate replacement, prefabricated Lego brick

## Abstract

Nowadays, more efforts towards sustainability are required from the concrete industry. Replacing traditional aggregates by recycled bottom ash (BA) from municipal solid waste incineration can contribute to this goal. Until now, only partial replacement has been considered to keep the concrete workability, strength and durability under control. In this research, the feasibility of a full aggregate replacement was investigated for producing prefabricated Lego bricks. It was found that the required compressive strength class for this purpose (C20/25) could be achieved. Nevertheless, a thorough understanding of the BA properties is needed to overcome other issues. As BA is highly absorptive, the concrete’s water demand is high. This workability issue can be dealt with by subjecting the fine BA fraction to a crushing operation to eliminate the porous elements and by pre-wetting the fine and coarse BA fractions in a controlled manner. In addition, a reactive NaOH washing is needed to avoid formation of longitudinal voids and the resulting expansion due to the metallic aluminum present in the BA. Regarding the long-term behavior, heavy metal leaching and freeze-thaw exposure are not problematic, though there is susceptibility to acetic and lactic acid attack and maybe increased sensitivity to alkali-silica reaction.

## 1. Introduction

Yearly, 3,331,000 tons of municipal solid waste are generated in Flanders. Around 880,000 tons are incinerated to gain energy and reduce the waste volume with 90% (Waste-to-Energy) [1,2]. The incineration results in bottom ash (BA), boiler ash and air pollution control residues. BA is mostly disposed of as landfill. The high waste volume, cost of landfill and lack of disposal sites force the industry to recycle the BA as secondary raw material [3]. This would also be more in agreement with European Directive 2008/98/EC [4], as it saves valuable natural resources and gives waste a financial value [5]. To obtain an end-of-waste status *cf.* ED 2008/98/EC [4], several criteria need to be fulfilled. The material should serve a purpose. The technical requirements and legislation of this purpose should be met. There needs to be a market for it and there should be no negative environmental effects [4]. In this context, companies involved in municipal solid waste incineration (MSWI) intend to recover the BA as aggregates for concrete. The successful production of so-called concrete Lego bricks is seen as the minimal aim. These are prefabricated unreinforced blocks which are used to build modular partition walls at industrial and rural sites. For several years now, the waste management company Indaver nv in Belgium has been trying to tune the properties of BA at its incineration plant to meet this goal. In the past, this already resulted in the publication of papers on the proper characterization and treatment of the Belgian BA (e.g., Vandecasteele *et al.* [6], Van Gerven *et al.* [7]).

According to literature, BA from Belgium [8,9], the Netherlands [10], France [11], Germany [5], Italy [3,12], Spain [2] and South-Korea [13] show quite some similarities in composition. They usually consist of bottle glass (around 15%, composed of Na_2_O, CaO, CaCO_3_ and SiO_2_ [2]), as well as metals (ferrous and non-ferrous, such as Al, Cu and Zn), ceramics (brick, plaster and mortar) and organic residues (bone fragments, charcoal, plant fibers and polymers). The latter elements have only a small contribution to the total mass (each with an amount of about 2%) [5,10]. Evidently, the variety in constituting materials implies a heterogeneous chemical composition of the BA: 30%–70% silica, 10%–15% aluminum oxide, 20% sodium oxide and 10%–15% calcium oxide. A smaller yet still important fraction of (heavy) metals is also present [5,10]. BA is lighter than gravel (2210 kg/m^3^ *vs.* 2470 kg/m^3^). The water absorption is 4–10 times the value of gravel (4%–10%). Both characteristics are due to a high porosity [3,11]. BA is highly angular and has a weaker abrasion strength than limestone [14]. BA concrete is usually characterized by a lower compressive strength, a lower Young’s modulus, a lower workability and a higher porosity than concrete with limestone as aggregate. Nevertheless, even without modifications and with full replacement of the aggregates by BA, a concrete strength class of C20/25 can be assured [15].

In the fresh state, extensive aggregate replacement by BA may have a negative effect on the concrete workability. This is mainly due to the higher water absorption of the BA. However, when using rather high water-to-cement (W/C) ratios, workability may not be a real issue. According to Pera *et al.*, a W/C ratio of 0.60–0.65 is ideal to achieve both an acceptable workability and a sufficient mechanical performance [11]. Moreover, the use of a superplasticizer (SP) can easily improve the workability if needed [16].

In hardened state, most damage phenomena are induced by the expansive reaction of metallic Al in an alkaline environment, which increases the porosity and reduces the strength. This effect exists for all hydroxide-forming elements present in the BA [5,11]. Alkali-silica reaction (ASR) is considered not detrimental because of the higher porosity of the BA concrete. A literature survey on other expected damage phenomena (e.g., freeze-thaw, acid attack, *etc.*) provides little or no information on the performance of BA concrete.

Literature on the leaching behavior of concrete containing BA from MSWI is rather scarce. Since the material is immobilized within the cementitious matrix of the concrete, leaching values are normally lower than when the individual grains are in direct contact with the leaching solution. Regarding the leaching of (heavy) metals, Sorlini and Ginés found values that meet the national criteria (Italy for Sorlini and Spain for Ginés) [17,18].

In the past, tests were mainly performed in view of a partial replacement of traditional aggregates by BA [10,11,16,17] and they included variations in cement type [17], W/C ratio [8,12], workability and compaction [8,19]. The concrete quality can also be improved by optimizing the BA properties using opto-mechanical glass separation [11], reactive washing with NaOH (=Lye treatment) [11], vitrification [12] and sintering [17].

## 2. Research Relevance and Methodology

In this research, the focus was on obtaining a qualitative concrete with replacement of traditional crushed limestone aggregates by BA for the production of prefabricated concrete products. The main novel character of the research lies in the fact that full instead of partial aggregate replacement is aimed for. The main benefit of such a practice is rather evident. If we are able to incorporate more BA from MSWI in concrete, less of the material will get landfilled, its valorization potential will increase significantly and the concrete industry will have to rely less on natural resources to ensure its production capacity. In other words, there will be considerable advantages from both an economic and environmental point of view. However, these benefits only exist if the production of this BA concrete is practically feasible in the casting stage and if the hardened concrete is sufficiently strong and durable for its intended field of application. Therefore, the following research approach was adopted. After careful characterization of the physical and chemical properties of the BA, several test mixtures with varying W/C ratios were made to find an optimal balance between workability and strength of the concrete. The effects of pre-treating the BA in various ways were carefully evaluated. A dedicated study of the expected long-term behavior for one potentially suitable mixture was done and a final optimized concrete mix design was proposed. Prefabricated Lego bricks were made using both mix designs and were compared with a Lego brick made of traditional concrete.

## 3. Materials and Methods

### 3.1. Concrete Constituents

The inert fraction of concrete traditionally consists of sand and aggregates. For the aggregates, it is often useful to use both a fine and a coarse fraction to achieve a better packing. In this research, the aim was a full replacement of the traditional fine and coarse aggregates by BA processed at the MSWI plant of Indaver nv. In terms of phase composition, this consisted of glass remains, a stony fraction (ceramics, porcelain, bricks, *etc.*), amorphous slags and some ferrous (e.g., steel) and non-ferrous (e.g., Al, Cu, Pb, Zn) metals that cannot be removed in the treatment installation. The glass remains (bottle glass, grains of glass with devitrification products) normally represent the largest fraction. The proportioning with the other fractions is somewhat seasonally bound.

Different types of fine and coarse BA aggregates were considered, *i.e.*, sieved and aged BA 0/6 and 2/6, crushed BA 0/6, sieved and washed BA 0/6 and 6/20, and crushed BA 6/20. The crushed and sieved and washed fractions originated from the same 6/50 fraction which was obtained after an initial processing stage at Indaver nv. The other fractions were the result of a natural aging process of a 2/6 batch. More details on how the 6/50 fraction and the aged 2/6 batch were obtained can be found in Vandecasteele *et al.* [6]. The crushing, sieving, washing and aging techniques that were applied afterwards to obtain the 0/6, 2/6 and 6/20 fractions were similar to the ones mentioned in that previous research. Crushing was done by means of a commercial mobile cone crusher that allows for setting the required maximum aggregate size. A mobile installation was also used for sieving the material to the desired sieve aperture. The material to be sieved was usually wet due to its outdoor unsheltered storage. No additional water was added during sieving. Washing operations were done in most cases with process water that resulted from drying the filter cake at the Indaver incineration plant. Logically, this process water contains fines that adhere somewhat to the BA. The aging process consisted of piling the BA on 5–10 m high heaps in open air on a paved floor at the incineration plant with the collection of percolating rainwater, exposing them to wind and rain.

The BA-based aggregates should replace the traditional crushed limestone 2/6 and 6/20 which are commonly used for concrete manufacturing in Belgium. The remaining portion of the inert fraction, *i.e.*, the sand, was a natural river sand 0/4.

Two different cement types were used for concrete manufacturing, either a traditional Portland cement CEM I 52.5 N MF or a low alkali (LA) high sulfate resistant CEM I 52.5 N HES LA HSR. The total cement content for each concrete composition amounted to 350 kg/m^3^.

The mixing water consisted of tap water at a temperature of 20 °C. The amount of water used for the concrete varied with the applied W/C ratio: 0.65, 0.60 or 0.55. When lowering the water content substantially, the use of a SP was often imperative. Initially, polycarboxylic ether-based MasterGlenium 51 con. 35% (dry matter mass percentage: 35%, density at 20 °C: 1100 kg/m^3^) was used for this purpose. Later on, a switch was made to polycarboxylic ether-based MasterGlenium ACE 30 con. 30% SPL (dry matter mass percentage: 35%, density at 20 °C: 1070 kg/m^3^) to reduce the delay in setting time.

### 3.2. Physical and Chemical Characterization of the Aggregates

#### 3.2.1. Water Absorption over 24 h and Mass Density

The water absorption over 24 h (WA_24_) of all studied aggregates was measured in accordance with NBN EN 1097-6 using a calibrated pycnometer. The apparent mass density ρ_a_ (kg/m^3^) and the relative mass density after oven drying ρ_rd_ (kg/m^3^) were determined in accordance with the same standard.

#### 3.2.2. Water Absorption as a Function of Time

To evaluate the water absorption as a function of time, a hydrostatic weighing technique similar to the one proposed by Garcia-Gonzalez *et al.* and Tegguer was applied [20,21]. Per aggregate type, a sample of at least 1000 g was inserted in a cylindrical strainer. This strainer was hung from a balance and submerged in a basin filled with demineralized water. The weight increase was monitored over a time period of 10 days.

#### 3.2.3. Particle Size Distribution

The particle size distributions of the aggregates and sand were determined *cf.* NBN EN 933-1. The sieving column consisted of standardized sieves with following apertures: 63, 45, 40, 31.5, 22.4, 20, 16, 14, 12.5, 10, 8, 6.3, 4, 2, 1, 0.5, 0.25, 0.125 and 0.063 mm. The cumulative percentage of material passing through each sieve was calculated to plot the particle size distributions.

#### 3.2.4. Aggregate Crushing Value

The strength of an aggregate can be characterized by its crushing value. In this research, this parameter—also known as the static compressive strength—was determined in accordance with Belgian standard NBN B11-205, for both the BA and the limestone aggregates. A cylindrical metal mold with an inner diameter of 150 mm was filled with a dried aggregate sample of a certain particle size range. After adding the sample, the mold was fitted with a no-friction plunger which was then loaded to 400 kN in 4 min. Before unloading again, this maximum load onto the sample was maintained for 2 min. Finally, the crushed fraction of the aggregate <2 mm was separated from the sample by means of a sieving operation. The mass ratio of the sample without the crushed fraction over the initial sample before loading counts as the static compressive strength of the aggregate. Normally, this test is to be conducted on coarse aggregate fractions only (minimum aggregate diameter: 6 mm). Nevertheless, the static compressive strengths of the fine BA and limestone aggregates were determined in the same manner. Per type of sample, the test was repeated three times.

#### 3.2.5. Chemical Composition

The chemical properties of the BA were determined using Inductively Coupled Plasma (ICP), Ion Chromatography (IC) and Flow Injection Mercury System (FIMS) techniques available at the laboratories of Indaver nv.

### 3.3. Concrete Mix Design and Production

Once the total cement content (=350 kg/m^3^) and the W/C ratio (0.65, 0.60 or 0.55) were set for the concrete compositions under investigation, the required amounts of sand, fine and coarse aggregates were determined. This was done by approximating the optimal particle size distribution curve of Fuller using the least-of-squares method. This approach normally results in a minimal spacing between the different particles of the inert fraction.

In the initial research stage only the fine aggregates were replaced by BA. In the next stage, the same was done for the coarse aggregates. As such, the individual effects of each BA could be studied unambiguously. In the final stage, both the fine and coarse limestone aggregates were replaced.

Concrete was produced in accordance with NBN EN 12390-2. First, the fine and coarse aggregates, the sand and the cement, were mixed for 1 min. Then, the water was added whereupon mixing continued for 2 min. After this step, the workability of the concrete was evaluated by means of the slump and flow test (NBN EN 12350-2, NBN EN 12350-5) and the required amount of SP was determined. This amount was added while mixing for another 2 min. The SP dosing step was repeated until the proper slump and flow class were obtained. Finally, the test samples were cast and optimally cured in a climate chamber at 20 ± 2 °C and 95% ± 5% relative humidity (RH).

### 3.4. Strength Performance

The compressive strength was determined in accordance with NBN EN 12390-2. This was done after seven days, 28 days and 56 days of optimal curing. Per testing age three cubic specimens (*n* = 3) with a 150 mm side were subjected to the test. The characteristic value of the compressive strength f_ck_ at 28 days was obtained in correspondence with EN 1990 *cf.* Equation (1).

(1) $$f_{\mathrm{ck}}=\bar{x}-k_{n}\times s\left(N/\mathrm{mm}²\right)$$

where x is the mean value of the compressive strength of n samples (N/mm^2^); k_n_ is a factor equal to 3.37 for *n* = 3 and a variation coefficient V_X_ unknown from prior knowledge; and s is the standard deviation on the individual values of the compressive strength of n samples. With the characteristic compressive strength known, the proper compressive strength class as specified in NBN EN 206-1 can be assigned.

### 3.5. Susceptibility to Expansion

The susceptibility to expansion was evaluated by monitoring the height of the cubic samples intended for the compressive strength tests as a function of time (after 1, 7, 28 and 56 days) and relative to the their original height just after casting (=150 mm). Note that this is only a rudimentary assessment approach to get a first idea on the expansive behavior. The change in length of only one cube side reflects the total expansion only to a certain extent since the initial early age expansion in other directions is hindered by the cubic mold. Moreover, the duration of dihydrogen gas generation after reaction with the metallic Al and the setting time of the concrete also govern the amount of expansion as well. Further investigation on these influencing parameters is certainly still necessary in the future.

### 3.6. Porosity

The porosity was determined using a vacuum saturation technique followed by hydrostatic weighing *cf.* NBN B 05-201. Since the amount of water absorbed during the vacuum saturation is mainly limited to the pores in contact with the outer sample surface, the resulting porosity only reflects the open/permeable porosity. Per concrete mixture, the test was performed on nine cylindrical samples (diameter: 100 mm, height: 50 mm) taken from cubes (side: 150 mm). After 28 days of optimal curing, the samples were weighed and dried at a temperature of 40 ± 5 °C until constant mass (Δm after 24 h < 0.1%). Next, the samples were placed in a normalized vacuum tank. A vacuum with a residual pressure of 2.7 kPa was applied for 2.5 h. While keeping this vacuum condition, water was introduced at a rate of 50 mm/h until complete immersion of the samples. Subsequently, the air pressure was restored and the samples were kept under water for 24 h. Afterwards, they were weighed hydrostatically as well as above water. After the weighing, they were dried at 105 ± 5 °C until constant mass (Δm after 24 h < 0.1%) whereupon the whole procedure was repeated. Then, the open/permeable porosity φ was calculated for the two pre-drying temperatures (Equation (2)).

(2) $$\varphi =\frac{m_{s}-m_{d}}{m_{s}-m_{l}}\times 100\left(\%\right)$$

where m_d_ is the oven dry mass after pre-drying at 40 °C or 105 °C (g); m_s_ is the water saturated mass after vacuum saturation (g); and m_l_ is the mass under water after vacuum saturation (g).

### 3.7. Long-Term Behavior

#### 3.7.1. Susceptibility to Leaching

After 28 days of optimal curing, six cubes (side: 100 mm) were conserved in a closed tank filled with demineralized water. Conform CMA/2/II/A, the volume of the reagents should be two to five times the sample volume. Conform NEN 7345, this is four times the sample volume. In this research, the latter volume ratio was chosen. A water sample was taken after 30 days of immersion and examined using ICP, IC and FIMS.

#### 3.7.2. Resistance to ASR

The susceptibility to ASR was evaluated using the modified Oberholster test *cf.* the Walloon technical guideline STM D424 ST6. This method was chosen because, in contrast with the original Oberholster test or South-African NBRI (National Building Research Institute) accelerated test method, it allows for a test directly on the concrete mixtures under investigation instead of on mortar bars containing the aggregates to be studied after an initial processing (crushing and sieving). Moreover, the modified Oberholster test holds the advantage of being a rather fast assessment technique (exposure period: 20 days) in comparison with some of the other methods designed for concrete (exposure periods of up to 12 months *cf.* RILEM TC 106-AAR [22]). Per mixture, six cylinders (diameter: 50 mm, height: 150 mm) taken from cubes (side: 150 mm) were tested. After 28 days of optimal curing the samples were immersed for 24 h in water at 80 °C. Afterwards, the samples were stored in a 40 g NaOH per L water solution at 80 °C. The expansion was continuously monitored on dial gauges in contact with the upper surface of the cylinders. Normally, an expansion exceeding 0.1% implies a potential ASR sensitivity.

#### 3.7.3. Resistance to Acid Attack

Six cylinders (diameter: 113 mm, height: 150 mm) were drilled and cut from six cubes (side: 150 mm). After 28 days, the cylinders were exposed to 15 cycles involving intermittent exposure to acid. One cycle started by keeping the samples 24 h in a dry environment (minimal 60% ± 5% RH) and continued with 24 h of immersion in an acid solution (30 g acetic acid and 30 g lactic acid per liter), all taking place at a temperature of 20 ± 2 °C and 60% ± 5% RH. After each drying period, the mass of the samples was determined. After the last cycle, the mass was determined once again. The damage was evaluated based on the observed mass decrease and visual appearance.

#### 3.7.4. Resistance to Freeze-Thaw Attack

Four cubes (side: 150 mm), optimally cured for 23 days, were subjected to a freeze-thaw test in compliance with NBN B15-231, which in turn refers to NBN B 05-203. The samples were submerged in demineralized water at a temperature of 20 ± 2 °C until constant mass (Δm 24 h < 0.05%) *cf.* NBN B 15-215. After saturation was observed, the samples were placed in a laboratory freezing chamber and were subjected to 14 freeze-thaw cycles which consisted of: (i) cooling down till 0 °C in 1–2 h; (ii) progressive freezing from 0 °C to −15 °C in 5 h (at a mean rate of 3 ± 0.5 °C/h and always in the range of 2–4 °C/h); (iii) maintaining a constant freezing temperature of −15 ± 2 °C for 10–11 h; (iv) defrosting in water; (v) keeping the samples in water until the end of the cycle at 24 h.

Two non-destructive test methods were used to evaluate the freeze-thaw resistance: a visual check-up and ultrasonic pulse velocity measurements using a commercial device. The visual damage assessment was done in accordance with NBN B 27-009/A1.

### 3.8. Microscopic Characterization on Thin Sections

To explain the porosity, a microscopic investigation on thin sections was necessary. These thin sections were prepared as follows: 45 × 30 × 15 mm^3^ prisms were cut from the concrete and 45 × 30 mm^2^ faces were glued onto glass slides with a thickness of 2.9 mm. Then, the samples were cut and polished until the height of the concrete specimens and the glass equaled 10.1 mm. In a next step, the samples were impregnated under vacuum with a fluorescent epoxy. After impregnation, the excess epoxy was ground away and an object glass was glued onto the polished surface. In a final step, the glass slides were cut off and the concrete samples on the object glasses were polished until thin sections with a 25 µm thickness were obtained. A cover glass was glued onto them for protection. All thin sections were examined with a Leica DM LP polarizing microscope. Images were taken with a Leica DFC295 camera in fluorescent light mode.

### 3.9. Optimization of the Bottom Ash

#### 3.9.1. Pre-Wetting

To limit the high water absorption during concrete mixing and the resulting workability issues, the BA could be pre-wetted. In total, three pre-wetting techniques were examined. All of them were based on their expected water absorption as a function of time (Section 3.2.2). The first technique focused on pre-wetting the BA to a level corresponding with the deflection point in the absorption curve. This level is met as soon as the initial high water uptake rate ends. The time needed for this counts as the required immersion time span of the aggregates prior to the concrete mixing. After immersion, a self-made strainer bucket was used to drain the water. Note that this method was only applied for the fine BA. The second and third methods also relate to the water absorption behavior as a function of time. For these two methods, the expected contact time with water was the decisive criterion and not the change in water absorption rate. As the concrete mixing process and specimen casting takes about 6 min after adding the water, it makes sense to pre-wet the BA with the amount of water that is expected to be absorbed within the 6 min timeframe. This can be done in two ways. Either the aggregates can be pre-wetted in the mixer (this second method is commonly used when manufacturing concrete with lightweight aggregates), or the additional amount of water can be added afterwards as a surplus to the mixing water (the third method). Both the fine and coarse BA were treated like this.

#### 3.9.2. Reactive Washing

The applied reactive washing procedure was based upon literature findings [23] and existing know-how at Indaver nv. To passivate Al inclusions, the BA was washed with 1 M NaOH. The BA was completely immersed in this solution for two weeks. The metallic Al reacts with the NaOH solution and forms non-active Al and H_2_. The mixture was stirred from time to time to ensure an optimal interaction between the BA and the solution and to guarantee a proper release of H_2_.

To evaluate the effectiveness of this reactive washing, the amount of reactive Al before and after the treatment was determined. Therefore, the BA samples were subjected to a test described in CUR recommendation 116 [24]. Per sample, the BA was combined with the NaOH solution in a closed system flask. The H_2_ being released was captured in a second flask initially full of water. Since a portion of the water is displaced by the H_2_, the amount of metallic Al can be calculated from Equation (3) after measuring the initial and final water weight.

(3) $$v_{H_{2}}=\frac{3}{2}\frac{m_{\mathrm{Al}}}{M_{\mathrm{Al}}}v_{0}\left(L\right)$$

where v_H2_ is the volume of entrapped H_2_ (L); M_Al_ is the molar mass of Al (27 g/mol); v_0_ is the molar volume (22.4 L/mol); and m_Al_ is the mass of reactive Al (g). Before applying the BA after the reactive washing in concrete, they were first rinsed with water until the pH had decreased sufficiently. For the fine and coarse BA fractions, the pH decreased from 12.8 to 9.0–10.0 and from 12.8 to 8.0, respectively.

### 3.10. Manufacturing of the Lego Bricks

Three 1600 × 800 × 400 mm^3^ Lego bricks were cast: one made of traditional concrete, one made of the initial BA concrete with full replacement of both the fine and coarse aggregate fractions and one made of the optimized BA concrete, also with full aggregate replacement. The mold for the Lego brick was provided by CB Recycling BV. The concrete was manufactured in a concrete mixer with a total volume capacity of only 200 L. As a consequence, the Lego bricks had to be cast in three subsequent stages. Each layer of concrete was carefully compacted using a vibration needle.

## 4. Results and Discussion

### 4.1. Physical and Chemical Characterization of the Bottom Ash

#### 4.1.1. Particle Size Distributions

**Figure 1:**
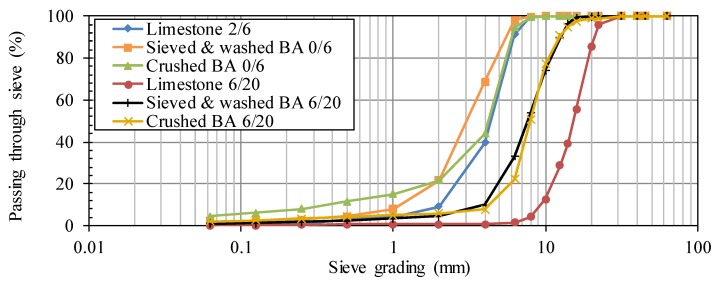
Influence of aggregate type (limestone *vs.* bottom ash (BA)), fraction (2/6 or 0/6 *vs.* 6/20) and BA pre-treatment (sieving and washing *vs.* crushing) on the particle size distribution.

The particle size distributions of crushed limestone 2/6 and BA 0/6 are within the same range (Figure 1). Only the fraction <1 mm is more present in BA 0/6, whereas the limestone has a steeper grading curve, indicating a pure and narrow range of grain diameters. The crushed fraction has more fines than the sieved and washed fraction. This is probably due to the dust generation during crushing.

When looking at the particle size distributions of the coarse fractions, a clear shift to the left can be observed between limestone 6/20 and BA 6/20. In accordance with the proper definitions for minimum and maximum aggregate size *cf.* NBN EN 12620, this BA fraction rather meets the requirements of an aggregate 2/14 instead of those of an aggregate 6/20. Comparison between the crushed and sieved and washed BA 6/20 reveals only very limited differences in particle size distribution.

Note that there are several factors with an influence on the difference in particle size distributions, e.g., the grading and strength of the original untreated material that is subjected to the processing as well as the effectiveness of the equipment used for crushing, sieving and washing.

#### 4.1.2. Aggregate Crushing Value

When making the comparison between the recorded aggregate crushing values or static compressive strengths for the sieved and washed and crushed BA and the limestone aggregates, it is immediately clear that the BA clearly shows a lower mechanical performance (Table 1).

**Table 1:** Influence of aggregate type (limestone *vs.* BA), fraction (2/6 *vs.* 6/10 and 6/20) and BA pre-treatment (sieving and washing *vs.* crushing) on the static compressive strength.

**Limestone 2/6**	**Limestone 6/20**	
Fraction 2/6	Fraction 6/10	Fraction 10/20
63.8% ± 0.9%	75.1% ± 0.7%	82.7% ± 0.3%
**Sieved & washed BA 0/6**	**Sieved and washed BA 6/20**	
Fraction 2/6	Fraction 6/10	Fraction 10/20
42.2% ± 0.2%	55.5% ± 1.0%	63.3% ± 0.5%
**Crushed BA 0/6**	**Crushed BA 6/20**	
Fraction 2/6	Fraction 6/10	Fraction 10/20
50.3% ± 0.6%	58.8% ± 0.3%	65.8% ± 0.8%

This statement holds true for both the fine and coarse aggregates. The difference ranges between 13.6% and 21.6%, depending on the aggregate type and fraction. It is worth mentioning that the crushed BA fractions are all characterized by a significantly higher static compressive strength than the sieved and washed fractions. It is likely that the crushing operations performed in the processing installations of Indaver nv reduced the weaker particles in size, to values lower than the particle sizes considered during the static compressive strength tests. Thus, only the stronger aggregates remain. In the case of the sieved and washed BA there was no elimination of the weaker elements as such.

#### 4.1.3. Mass Density and Water Absorption over 24 h

From the apparent mass densities recorded (Table 2), it is clear that BA is a lighter aggregate than the limestone. This statement holds true for both the fine and the coarse fractions. The relation between the surface dry mass density ρ_rd_ and the apparent mass density ρ_a_ gives an indication of the material’s porosity. As such, the third column of the table clearly shows that the BA is more porous than the reference material. In case of the fine fraction, the crushed BA has a significantly lower porosity than the sieved and washed BA. On the other hand, the porosities of the crushed and sieved and washed coarse fractions are not significantly different from each other.

**Table 2:** Influence of aggregate type (limestone *vs.* BA), fraction (2/6 or 0/6 *vs.* 6/20) and BA pre-treatment (sieving and washing *vs.* crushing) on the apparent mass density (ρ_a_), surface dry mass density (ρ_rd_) and water absorption after 24 h (WA_24_).

Aggregate Type	ρ_a_ (kg/m^3^)	ρ_rd_ (kg/m^3^)	(ρ_a_−ρ_rd_)/ρ_a_ × 100 (%)	WA_24_ (%)
**Limestone 2/6**	2750 ± 31	2644 ± 37	3.85 ± 0.28	1.5 ± 0.1
Sieved and washed BA 0/6	2246 ± 13	1793 ± 13	20.17 ± 0.44	11.2 ± 0.3
Crushed BA 0/6	2536 ± 6	2144 ± 11	15.46 ± 0.59	7.2 ± 0.3
**Limestone 6/20**	2762 ± 18	2710 ± 16	1.87 ± 0.06	0.7 ± 0.0
Sieved and washed BA 6/20	2649 ± 7	2285 ± 11	13.72 ± 0.20	6.0 ± 0.1
Crushed BA 6/20	2697 ± 22	2329 ± 23	13.65 ± 0.25	5.8 ± 0.1

More or less similar conclusions can be drawn from the water absorption after 24 h (Table 2: WA_24_). This parameter is to a high extent correlated to the ratio of apparent and surface dry mass density. It is immediately clear that the BA has a higher inherent water absorption than the corresponding reference limestone aggregates. The value is about eight times the value of crushed limestone. Fine BA obtained by crushing has a substantially lower water absorption than the sieved and washed fine BA. Its value is comparable to the ones of the coarse BA. As indicated by the static compressive strength values (Section 4.1.2), crushing removes more of the weaker elements in the fine BA. These are probably the more porous elements responsible for the high water absorption. Thus, the crushing operation as pre-treatment seems beneficial for the fine fraction in order to control that property. In general, the fine fraction still absorbs more water than the coarse one. This is a recurring phenomenon for all types of aggregate materials due to their higher surface area. Nevertheless, in case of a highly absorptive BA aggregate, this particular behavior of the fine fraction may result in workability issues when used in concrete (see Section 4.2.1).

#### 4.1.4. Water Absorption as a Function of Time

**Figure 2:**
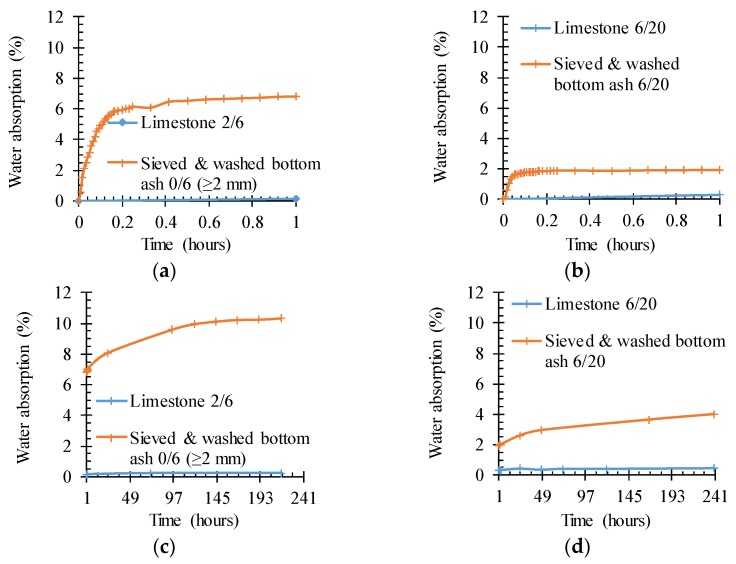
The higher water absorption as a function of time for sieved and washed BA 0/6 (≥2 mm) and 6/20 in comparison with limestone 2/6 and 6/20. (**a**) fine, between 0 and 1 h; (**b**) coarse, between 0 and 1 h; (**c**) fine, between 1 and 241 h; (**d**) coarse, between 1 and 241 h.

Figure 2 shows the water absorption as a function of time. The reference limestone aggregate materials barely absorb water within the considered timeframe of around 240 h. The fine and coarse BA fraction are much more absorptive and show a clear deflection point after around 2.5 h of immersion in water. At this point, the fine fraction has already reached 70% of its total absorption capacity, while the coarse BA reaches 50% of its capacity. Note that the fine BA already absorbs over 60% of its total absorption capacity within the first 15 min which is a very relevant timeframe within the concrete manufacturing process.

#### 4.1.5. Chemical Composition

The presence of (heavy) metals is of relevance in view of the concrete performance (e.g., the susceptibility to expansion, see Section 4.2.3) and leaching-related environmental problems (see Section 4.4.1). The chemical composition was determined for the sieved and washed BA 0/6, the crushed BA 0/6 and the sieved and washed BA 6/20 (Table 3). Unfortunately, there was no crushed BA 6/20 available at the time of the chemical analysis at the laboratories of Indaver nv. The (heavy) metal content is higher for sieved and washed BA 0/6 than for crushed BA 0/6 and sieved and washed BA 6/20. Also, the amount of Al is lower in case of the latter two BA aggregates, which indicates a lower susceptibility to Al-induced expansion. On the other hand, Fe, Cu and Zn are less present in the sieved and washed 0/6 fraction. This either indicates that these elements were less present in the MSW that was incinerated to obtain this batch of BA or that the separation of these ferrous and non-ferrous elements was more successful.

**Table 3:** Influence of the fraction (0/6 *vs.* 6/20) and pre-treatment (sieving and washing *vs.* crushing) on the chemical composition of the BA, especially in view of the (heavy) metal content.

Element	Sieved and Washed BA 0/6	Crushed BA 0/6	Sieved and Washed BA 6/20
Al (μg/L)	31,590	5757	3920
Ba (μg/L)	61	148	58
Cu (μg/L)	61	213	117
Zn (μg/L)	13	1332	167
Fe (mg/L)	<0.4	3.0	0.7
Ca (mg/L)	156	137	146
K (mg/L)	45	51	58
Na (mg/L)	85	51	58

### 4.2. Concrete Mixtures

#### 4.2.1. Mixture Proportions and Fresh State Properties

Lowering the W/C ratio normally results in the application of a higher SP dosage to achieve a sufficient workability (slump class S3–S4). For the reference concrete (Table 4: mixtures REF1–REF4), SP only needed to be added in case of a W/C ratio of 0.55. In case of a replacement of the fine limestone 2/6 by fine BA (Table 4: mixtures BA1–BA12 and BA24), the concrete workability was more problematic. Much higher SP dosages (up to 8 mL/kg cement) were necessary and still it remained difficult to achieve the same workability as for the reference concrete, even if the W/C ratio amounted to 0.60 or 0.65. The use of crushed BA instead of sieved and washed BA turned out less problematic somehow. The insufficient workability can be attributed to the fact that the BA has a high porosity and will thus absorb a lot of water in the beginning. Nevertheless, when also considering mixtures BA13–BA16 with replacement of only the coarse fraction by BA 6/20 (Table 4), which is also quite porous (see Table 2), workability was much less of an issue. Still, when considering full replacement of the limestone by fine and coarse BA (Table 4: mixtures BA17–BA23 and BA25–BA28), the detrimental effect of the fine fraction kept on dominating the workability and the required SP dosage. For the W/C ratio of 0.55, this dosage amounted to no less than 36 mL/kg cement, which is far above the dosage allowed for that type of SP. Moreover, the high dosages would increase the price of these mixtures for rather low-value applications (*i.e.*, prefabricated Lego bricks) quite dramatically.

#### 4.2.2. Strength Performance

For the reference concrete (REF1–REF4), adequate strength results (>33 N/mm²) are already obtained after only seven days of curing (Table 5), including for the highest W/C ratio considered (=0.65). For the lower W/C ratios of 0.60 and 0.55, these initial strength results amount to no less than 38 and 44 N/mm², respectively. This strength performance is still bound to increase because of the still-ongoing hydration of the cement. The most significant strength increase could be observed between 7 and 28 days of curing. As a consequence, the 28-day compressive strength class of this traditional concrete type is at least C30/37, which makes it very suitable for prefabricated Lego brick production, which requires a strength class of only C20/25.

In case only the fine aggregate fraction has been replaced with BA (Table 5: mixtures BA1–BA12 and BA24), the concrete performed worse in terms of compressive strength. Still, the 28-day strength class was at least C20/25 in most cases. Mixture BA10 was the only exception. This can mainly be attributed to the 20 min immersion of the fine BA in water prior to the mixing process. The pre-treatment with water was very beneficial for the concrete workability (Table 4: slump class S4, without SP addition). However, the strength decrease caused by it (Table 5: C16/20) proved rather unacceptable given the intended purpose of the concrete. Lowering the W/C ratio from 0.65 over 0.60 to 0.55 obviously turned out beneficial for the strength performance. For these W/C ratios, a 20 min immersion in water as a pre-treatment still resulted in concrete with strength class C20/25 (Table 5: mixtures BA11–BA12).

The replacement of only the coarse limestone by BA 6/20 (Table 5: mixtures BA13–BA15) clearly had a less detrimental effect on the strength. Just as for the reference concrete mixtures, the compressive strength is already higher than 30 N/mm^2^ after only seven days of optimal curing. Their 28-day strength class was at least C25/30 and can reach C30/37 for W/C ratios of 0.60 and 0.55. As such, a more or less similar strength performance to the reference concrete can be assumed. Only the strength gain between 28 days and 56 days of optimal curing is more pronounced for the BA concrete.

**Table 4:** Influence of the mixture proportions (ratio sand, fine and coarse aggregates, water-to-cement (W/C) ratio and superplasticizer (SP) dosage), replacement of limestone by BA and BA pre-treatment on the concrete workability.

Mixture	Natural Aggregates			BA		Water	Cement CEM I 52.5 N	SP	W/C Ratio	Workability	
	Sand 0/4	Limestone 2/6	Limestone 6/20	BA 0/6/BA 2/6	BA 6/20					Slump	Flow
	(kg/m^3^)	(kg/m^3^)	(kg/m^3^)	(kg/m^3^)	(kg/m^3^)	(kg/m^3^)	(kg/m^3^)	(mL/kg Cement)	-	-	-
REF1	439.00	596.00	695.00	-	-	227.50	350.00	-	0.65	S4	-
REF4	439.00	596.00	695.00	-	-	227.50	350.00	-	0.65	S4	F5
REF3	451.00	613.00	714.00	-	-	210.00	350.00	-	0.60	S4	F5
REF2	464.00	627.00	733.00	-	-	192.50	350.00	1 ^a^	0.55	S3	F4
BA2	333.00	-	674.00	664.00 ^b^	-	227.50	350.00	3 ^c^	0.65	S3	-
BA3	282.00	-	700.00	690.00 ^b, d^	-	227.50	350.00	3 ^c^	0.65	S3	-
BA4	471.00	^−^	686.00	519.00 ^e^	-	227.50	350.00	2 ^c^	0.65	S4	-
BA6	436.00	-	717.00	525.00 ^e^	-	227.50	350.00	1 ^c^	0.65	S2	-
BA7	436.00	-	717.00	525.00 ^e^	-	227.50	350.00	1 ^a^	0.65	S2	-
BA10	436.00	-	717.00	525.00 ^b^	-	227.50 ^f^	350.00	-	0.65	S4	-
BA1	342.00	-	350.00	683.00 ^b^	-	210.00	350.00	2 ^c^	0.60	S1	-
BA5	342.00	-	665.00	683.00 ^b^	-	210.00	350.00	8 ^c^	0.60	S4	-
BA8	448.00	-	737.00	539.00 ^e^	-	210.00	350.00	3 ^a^	0.60	S2	-
BA11	448.00	-	737.00	539.00 ^b^	-	210.00 ^f^	350.00	-	0.60	S3	-
BA24	397.00	-	703.00	557.00 ^g^	-	210.00	350.00	-	0.60	S4	F4
BA9	460.00	-	757.00	554.00 ^e^	-	192.50	350.00	6 ^a^	0.55	S3	-
BA12	460.00	-	757.00	554.00 ^b^	-	192.50 ^f^	350.00	-	0.55	S2	-
BA13	613.00	444.00	-	-	633.00 ^h^	227.50	350.00	-	0.65	S3	F4
BA14	630.00	456.00	-	-	650.00 ^h^	210.00	350.00	-	0.60	S3	F4
BA16	617.00	540.00	-	-	594.00 ^i^	210.00	350.00	-	0.60	S4	F3
BA15	647.00	469.00	-	-	668.00 ^h^	192.50	350.00	6 ^a^	0.55	S3	F4
BA17	517.00	-	-	347.00 ^j^	797.00 ^h^	227.50	350.00	6 ^a^	0.65	S3	F4
BA20	517.00	-	-	347.00 ^j^	797.00 ^h^	227.50	350.00	5 ^a^	0.65	S3	F4
BA21	517.00	-	-	347.00 ^j^	797.00 ^h^	227.50 ^k^	350.00	-	0.65	S4	F5
BA22	517.00	-	-	347.00 ^j^	797.00 ^h^	227.50 ^l^	350.00	-	0.65	S2	F4
BA23	517.00	-	-	347.00 ^j^	797.00 ^h^	227.50	350.00 ^m^	3 ^a^	0.65	S2	F4
BA26	488.00	-	^−^	324.00 ^j^	793.00 ^h*^	227.50 ^n^	350.00	-	0.65	S4	F4
BA27	488.00	-	-	324.00 ^j^	793.00 ^h*^	227.50 ^o^	350.00	6 ^a^	0.65	S2	F1
BA28	543.00	-	-	343.00 ^j, p^	727.00 ^h, p^	227.50	350.00	2 ^a^	0.65	S2	F4
BA18	531.00	-	-	357.00 ^j^	819.00 ^h^	210.00	350.00	10 ^a^	0.60	S2	F3
BA25	547.00	-	-	400.00 ^g^	717.00 ^i^	210.00	350.00	5 ^a^	0.60	S3	F4
BA19	545.00	-	-	366.00 ^j^	841.00 ^h^	192.50	350.00	36 ^a^	0.55	S3	F2
Lego brick 1	488.00	-	-	366.00 ^j^	808.00 ^h*^	227.50	350.00	10 ^a^	0.65	S2	F1
Lego brick 2	439.00	596.00	695.00	-	-	227.50	350.00	-	0.65	S3	F5
Lego brick 3	667.00	-	-	382.00 ^g, p^	566.00 ^h*^	227.50 ^n^	350.00	-	0.65	S4	F5

Notes: ^a^ MasterGlenium ACE; ^b^ BA 0/6 sieved and aged; ^c^ MasterGlenium 51; ^d^ glass and metals removed; ^e^ BA 2/6 sieved and aged; ^f^ BA submerged for 20 min in water; ^g^ BA 0/6 crushed; ^h^ BA 6/20 sieved and washed; ^i^ BA 6/20 crushed; ^j^ BA 0/6 sieved and washed; ^k^ wetted before mixing: 5 m% BA 0/6 and 2 m% BA 6/20; ^l^ extra water added to mixing water: 5 m% BA 0/6 and 2 m% BA 6/20; ^m^ CEM I 52.5 N HES LA HSR; ^n^ wetted before mixing: 2.5 m% BA 0/6 and 1 m% BA 6/20; ^o^ extra water added to mixing water: 2.5 m% BA 0/6 and 1 m% BA 6/20; ^p^ after reactive washing with NaOH; ^*^ indicates a new delivery.

**Table 5:** Influence of W/C ratio, replacement of limestone by BA and pre-treatment of the BA on the compressive strength after 7, 28 and 56 days and the strength class.

Mixture	W/C (-)	Mix Details	f_c 7 days _(N/mm^2^)	f_c 28 days _(N/mm^2^)	f_c 56 days_ (N/mm^2^)	Strength Class (-)
REF1	0.65	-	33.49 ± 1.38	41.00 ± 0.43	44.85 ± 0.41	C30/37
REF4	0.65	-	33.61 ± 0.42	40.45 ± 0.61	41.72 ± 0.92	C30/37
REF3	0.60	-	38.54 ± 0.81	48.60 ± 1.14	49.37 ± 1.18	C30/37
REF2	0.55	^a^	44.29 ± 0.55	52.51 ± 1.43	55.15 ± 0.75	C35/45
BA2	0.65	^b, c^	23.43 ± 0.87	29.18 ± 0.71	31.26 ± 1.01	C20/25
BA3	0.65	^b, c, d^	29.69 ± 1.16	32.85 ± 0.41	33.26 ± 0.54	C25/30
BA4	0.65	^c, e^	32.75 ± 1.26	37.23 ± 0.29	38.32 ± 1.07	C25/30
BA6	0.65	^c, e^	28.62 ± 1.53	34.49 ± 0.46	38.30 ± 1.32	C25/30
BA7	0.65	^a, e^	28.69 ± 1.21	34.73 ± 0.42	40.46 ± 0.91	C25/30
BA10	0.65	^b, f^	18.88 ± 0.45	22.98 ± 0.68	26.66 ± 0.53	C16/20
BA1	0.60	^b, c^	25.52 ± 0.63	31.35 ± 0.81	34.95 ± 0.69	C20/25
BA5	0.60	^b, c^	30.39 ± 1.54	36.51 ± 1.54	38.10 ± 0.50	C25/30
BA8	0.60	^a, e^	33.40 ± 0.46	37.81 ± 0.53	42.16 ± 1.42	C25/30
BA11	0.60	^b, f^	20.82 ± 0.80	25.73 ± 0.14	29.24 ± 0.34	C20/25
BA24	0.60	^g^	30.34 ± 0.32	36.93 ± 1.38	39.10 ± 2.05	C25/30
BA9	0.55	^a, e^	36.62 ± 0.97	42.15 ± 1.28	43.20 ± 1.26	C30/37
BA12	0.55	^b, f^	22.35 ± 0.67	27.71 ± 0.70	31.83 ± 0.67	C20/25
BA13	0.65	^h^	34.40 ± 0.56	40.90 ± 2.13	46.32 ± 1.24	C25/30
BA14	0.60	^h^	37.40 ± 0.71	42.82 ± 1.35	50.30 ± 0.84	C30/37
BA16	0.60	^i^	35.23 ± 1.01	39.85 ± 0.86	47.42 ± 0.80	C25/30
BA15	0.55	^a, h^	38.34 ± 0.93	40.97 ± 0.53	47.73 ± 2.08	C30/37
BA17	0.65	^a, h, j^	32.93 ± 1.74	38.72 ± 0.61	40.18 ± 1.30	C25/30
BA20	0.65	^a, h, j^	26.26 ± 0.71	31.51 ± 1.10	33.15 ± 0.55	C20/25
BA21	0.65	^h, j, k^	22.03 ± 0.70	29.86 ± 0.92	30.55 ± 0.51	C20/25
BA22	0.65	^h, j, l^	25.05 ± 0.13	33.11 ± 1.06	34.80 ± 1.71	C20/25
BA23	0.65	^a, h, j, m^	26.67 ± 0.30	34.81 ± 0.21	34.92 ± 0.74	C25/30
BA26	0.65	^h*, j, n^	26.23 ± 0.74	32.47 ± 1.17	34.59 ± 0.10	C20/25
BA27	0.65	^a, h*, j, o^	21.74 ± 0.37	26.03 ± 0.94	28.11 ± 0.23	C20/25
BA28	0.65	^a, h, j, p^	30.23 ± 1.14	37.02 ± 0.46	38.62 ± 0.40	C25/30
BA18	0.60	^a, h, j^	28.38 ± 0.66	34.53 ± 0.76	33.67 ± 1.91	C25/30
BA25	0.60	^a, g, i^	32.15 ± 0.18	37.38 ± 0.95	38.98 ± 0.88	C25/30
BA19	0.55	^a, h, j^	29.72 ± 2.90	35.80 ± 1.11	36.18 ± 4.37	C25/30

Notes: ^a^ MasterGlenium ACE; ^b^ BA 0/6 sieved and aged; ^c^ MasterGlenium 51; ^d^ glass and metals removed; ^e^ BA 2/6 sieved and aged; ^f^ BA submerged for 20 min in water; ^g^ BA 0/6 crushed; ^h^ BA 6/20 sieved and washed; ^i^ BA 6/20 crushed; ^j^ BA 0/6 sieved and washed; ^k^ wetted before mixing: 5 m% BA 0/6 and 2 m% BA 6/20; ^l^ extra water added to mixing water: 5 m% BA 0/6 and 2 m% BA 6/20; ^m^ CEM I 52.5 N HES LA HSR; ^n^ wetted before mixing: 2.5 m% BA 0/6 and 1 m% BA 6/20; ^o^ extra water added to mixing water: 2.5 m% BA 0/6 and 1 m% BA 6/20; ^p^ after reactive washing with NaOH; ^*^ indicates a new delivery.

In case of a full replacement of both the fine and coarse aggregate fraction by BA (Table 5: mixtures BA17–BA23 and BA25–BA28), the earlier observed rather negative strength effects inherent to the fine fraction keep on dominating the overall strength performance of the concrete. Nevertheless, it remains possible to achieve a strength class for the concrete of at least C20/25, even when additional water is added to the BA before mixing to achieve a sufficiently workable concrete without adding SP (mixtures BA21–BA22). Lowering the W/C ratio from 0.65 to 0.60 and 0.55 makes it easier to obtain a strength class of C25/30.

Note that the lower strength performance of the BA concrete can mainly be attributed to the lower aggregate crushing value or static compressive strength of the BA aggregates (Section 4.1.2) and the higher porosity of the material. A more in-depth evaluation of the latter property follows in Section 4.2.3.

#### 4.2.3. Susceptibility to Expansion

**Figure 3:**
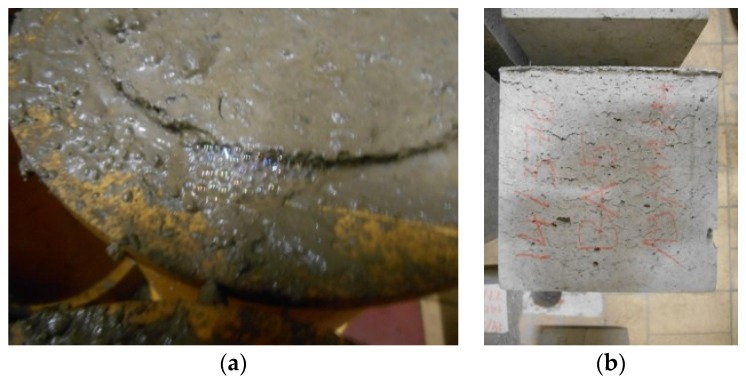
Reactive Al induced expansion near the troweled surface of a concrete cylinder (**a**); longitudinal expansion voids observed on a concrete test cube (**b**) when containing BA.

All concrete specimens with BA showed signs of expansion (Figure 3a). This behavior is attributed to the presence of metallic Al in the BA. When placed in an alkaline environment (pH > 7), the metallic Al dissolves and emits dihydrogen gas [11]. Obviously, the cement matrix (pH 13) provides these required alkaline conditions. The dihydrogen-induced expansion leads to the formation of longitudinal voids, which were, in this study, mainly concentrated near the troweled surface of the test samples and perpendicular to the casting direction (Figure 3b).

**Figure 4:**
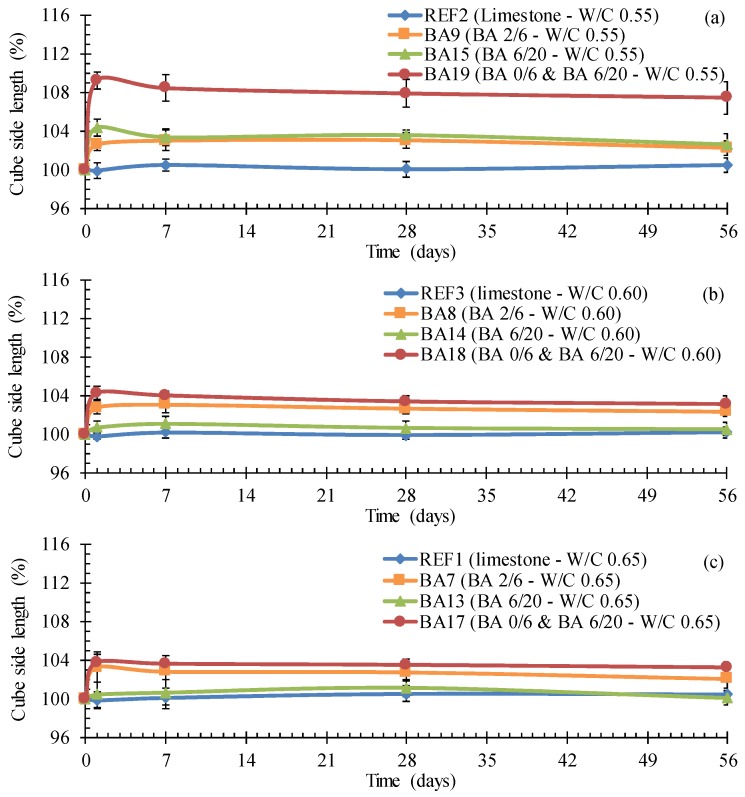
The higher susceptibility of BA concrete toward expansion in comparison with limestone concrete, an expansion which decreases with increasing W/C ratio 0.55 (**a**); 0.60 (**b**); 0.65 (**c**).

There is a clear difference in expansive behavior between the reference concrete and concrete in which the fine and/or the coarse aggregate fractions were replaced with BA. For all three W/C ratios (Figure 4a: 0.55, Figure 4b: 0.60, Figure 4c: 0.65), the expansive behavior of the BA concrete is evident. Whereas the reference concrete never shows a significant change in length for the distance between the bottom and the troweled surface of the test cubes, the concrete with replacement of the fine aggregate by BA is characterized by an expansion of a little less than 4%. Replacement of only the coarse aggregate fraction by BA 6/20 gives less expansion (around 1%), be it only for W/C ratios of 0.65 and 0.60. For a W/C ratio of 0.55, the expansion also equaled around 4%. The recorded expansion in case of a full replacement of the limestone by the appropriate proportioning of fine and coarse BA represents an accumulative effect of the two alternative aggregate fractions. This accumulative effect is the most pronounced for the lowest W/C ratio considered, *i.e.*, 0.55.

The BA concrete with the highest W/C ratio probably shows less expansion because it requires much less SP. The presence of SP usually tends to postpone the time of setting. This means that there is more time for the dihydrogen-induced longitudinal voids to form and, thus, also more time for the resulting expansion to develop. Nevertheless, further testing is still needed to find further confirmation for this.

#### 4.2.4. Selection of the Most Suitable Test Mixture

The selection of the most suitable BA concrete mixture for further testing was mainly governed by the achievable balance between workability (without extensive use of expensive SP) and strength performance. Compressive strength testing revealed that even with a W/C ratio of 0.65, the minimum required strength class for prefabricated Lego bricks (C20/25) could still be met (Section 4.2.2). Knowing this, it seems no problem to stick to this high W/C ratio as it also helps to compensate for the workability issues inherent to the presence of mainly the fine BA (Section 4.2.1). Sieved and washed BA were used for mixture BA20. This particular pre-treatment does not result in a significantly higher strength or resistance to expansion as opposed to the crushed BA. Nevertheless, sieving and washing normally leads to a somewhat lower workability than crushing. Still, the former pre-treatment was chosen since a crushing of the aggregates is more energy intensive and it does not improve the concrete workability that much. Instead, a suitable pre-wetting procedure for the BA was investigated in order to lower the required SP dosage for an adequate workability (see Section 4.5.1). Mixture BA20, still without pre-wetting of the BA aggregates, was manufactured for a further in-depth evaluation of the concrete mainly regarding its porosity (Section 4.3) and expected behavior in the long term (Section 4.4). Within Section 4.5.2, an additional reactive washing step was considered for the BA to overcome the expansion problem.

### 4.3. Porosity

From the vacuum saturation experiments, it can be concluded that the BA concrete is clearly more porous than the reference concrete (Table 6). The pronounced porosity difference between the BA and the reference concrete exists both after pre-drying at 40 °C and 105 °C.

**Table 6:** The higher open/permeable porosity of concrete BA20 *vs.* REF4 both after pre-drying at 40 °C (capillary porosity) and 105 °C (total porosity) and their similar calculated gel porosity.

Mixture	Capillary Porosity φ (%) after Pre-Drying at (40 ± 5) °C	Total Porosity φ (%) after Pre-Drying at (105 ± 5) °C	Gel Porosity φ (%)
REF4	11.1 ± 0.4	16.1 ± 0.6	5.1 ± 0.4
BA20	17.6 ± 0.4	22.5 ± 0.5	4.9 ± 0.1

In literature, the porosity obtained after pre-drying at 40 °C is said to correspond with the capillary porosity (pore diameter: 1–10 µm), while the difference in porosity obtained after pre-drying at 105 °C and 40 °C should represent the gel porosity (pore diameter: 10 nm) [25]. The capillary pores are the result of the fact that water and cement in unbound conditions take a larger volume than the hydrated cement. The gel pores relate to the gel water which is physically adsorbed by the hydration products and the monomolecular water film in between the flat crystallization products of interlayer water. When considering the theory of the capillary and gel porosity, one could state that the gel porosity is equal for both mixtures, while the capillary porosity is significantly different. However, since for both mixtures the same amount of water and cement was used, there is no reason to conclude that the capillary porosity should be different. Now, since the BA aggregates absorb a lot of water during the mixing process, it can be stated that the BA concrete has a lower effective water/cement ratio and thus also a lower capillary porosity. The increase in porosity after pre-drying at 40 ± 5 °C should be assigned to the existence of other voids, *i.e.*, the longitudinal expansion voids at the surface and the pores in the aggregates, if capillary pores reach these aggregates. The expansion voids will not directly attract water as their size (around 100 μm in width) does not really accommodate capillary suction. Still, these voids enlarge the possible water penetration surface from which more capillary pores can be reached. If a porous aggregate is in contact with them, an extra amount of water would be stored in the concrete as well.

Note that the capillary pores and the longitudinal expansion voids are influencing the strength and permeability of the concrete. The higher porosity after pre-drying at 40 ± 5 °C is a measure for the extra voids at the surface, but not of the inner structure. However, one could state that if these voids could be minimized, permeability will be diminished and strength will increase.

**Figure 5:**
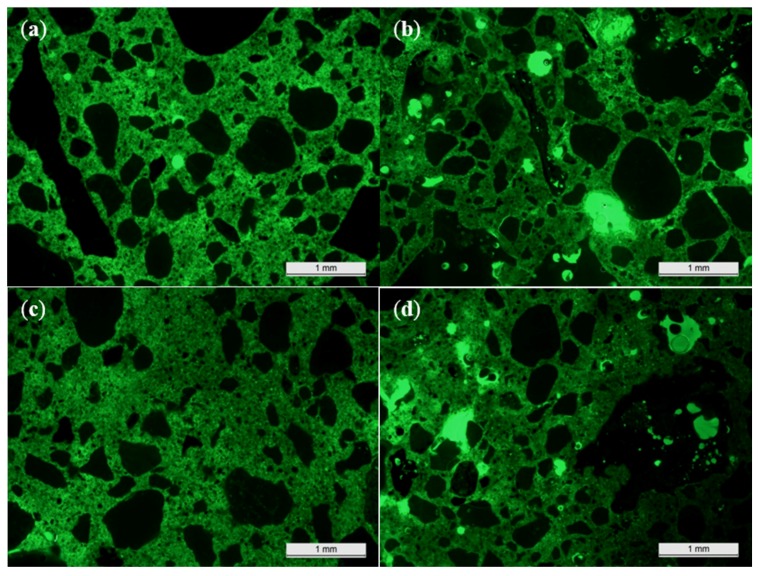
The darker green color of the thin sections of concrete BA20 *vs.* limestone concrete REF4 in fluorescent light mode, indicating a lower effective W/C ratio of the former. (**a**) REF4, thin section 1; (**b**) BA20, thin section 1; (**c**) REF4, thin section 2; (**d**) BA20, thin section 2.

For a better insight on the inner porosity, a microscopic analysis was performed as well (Figure 5).

When analyzing the thin sections in fluorescent light mode, it can be seen that the cement matrix of the BA concrete has a darker green color than the one of the reference concrete. This normally indicates that the effective W/C ratio of the BA concrete is lower. The concrete’s effective water content was also calculated. In accordance with NBN EN 206-1, this is the reduced amount of mixing water available given the water absorption (WA_24_) of the concrete’s inert fraction. The calculation revealed that when aiming at a theoretical W/C ratio of 0.65, the effective W/C ratio due to the high water absorption of the BA would only be 0.56. It also means that the higher open/permeable porosity for the BA mixture after pre-drying at 40 °C cannot be assigned to the presence of more capillary pores. Hence, it must indeed be caused by the presence of the longitudinal expansion voids and the highly absorptive aggregates.

### 4.4. Long-Term Behavior

#### 4.4.1. Susceptibility to Leaching

Leaching values stayed below the limiting values imposed by the Flemish VLAREMA standards for the normalized elements, except for bromine (Table 7). Copper was much more present in the leaching solution of the BA concrete as opposed to the one of the reference concrete (28 µg/L *vs.* <6 µg/L). Still, the measured concentration was acceptable (<80 µg/L). There is a two-fold explanation for the adequate leaching behavior. First of all, the BA itself already meets the VLAREMA criteria. Leaching components usually come from the bottle glass and metal fragments present in the BA, but in this study these fractions apparently had no problematic effects. Secondly, once in concrete they are immobilized in the cement matrix which reduces the leaching even more. Leaching data published by Vandecasteele *et al.* on the original non-processed BA 6/50 further confirm both effects [6]. The highest leaching value was recorded for Al (5302 μg/L), for which there are no VLAREMA criteria. It was about the 10-fold of the Al leaching value of the reference concrete.

**Table 7:** Leaching values for concrete BA20 and REF4 relative to demineralized water, indicating a fulfillment of most VLAREMA criteria, yet with a much higher Al leaching value for BA20.

Normalized	BA20	REF4	Demineralized Water	Vlarema Criterion	Non-Normalized	BA20	REF4	Demineralized Water
As (μg/L)	<12	<12	<12	80	Al (μg/L)	5302	620	<100
Ba (μg/L)	<4	<4	<4	200	B (μg/L)	480	182	49
Cd (μg/L)	<2	<2	<2	3	Mn (μg/L)	<6	<6	<6
Co (μg/L)	<2	<2	<2	50	Tl (μg/L)	<15	<15	<15
Cr (μg/L)	51	53	<4	260	Fe (mg/L)	<0.4	<0.4	<0.4
Cu (μg/L)	28	<6	<6	80	Ca (mg/L)	15	6.4	<1
Mo (μg/L)	49	31	<10	3000	K (mg/L)	169	136	<1
Ni (μg/L)	<2	<2	<2	75	Mg (mg/L)	<1	<1	<1
Pb (μg/L)	<20	<20	<20	130	Na (mg/L)	160	96	<1
Sb (μg/L)	<12	<12	<12	100	P (mg/L)	<0.02	0.06	<0.02
Se (μg/L)	<10	<10	<10	200	S (mg/L)	8.8	10.7	<0.02
Sn (μg/L)	<10	<10	<10	100	NO_2_ (mg/L)	<1	<1	<1
V (μg/L)	165	169	<10	250	NO_3_ (mg/L)	<1	<1	<1
Zn (μg/L)	<8	<8	<8	280	PO_4_ (mg/L)	<5	<5	<5
F (mg/L)	<5	<5	<5	5.5	I (mg/L)	<5	<5	<5
Cl (mg/L)	<100	<100	<100	100				
Br (mg/L)	<4	<4	<4	2				
SO_4_ (mg/L)	24	30	<10	220				
Hg (μg/L)	<1	<1	<1	2				

#### 4.4.2. Resistance to ASR

**Figure 6:**
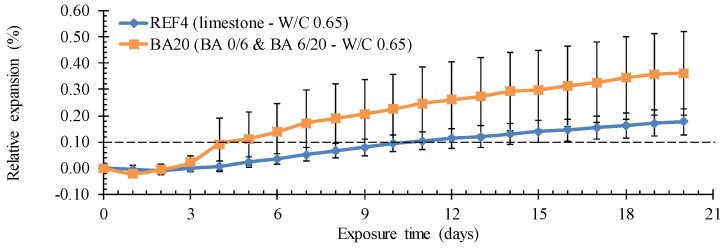
Higher mean relative expansion of concrete BA20 *vs.* limestone concrete REF4 after the modified Oberholster test.

After the modified Oberholster test, substantial expansion and cracking were observed for the BA concrete. The length increase was around twice the value of the reference concrete (Figure 6: 0.36% ± 0.16% *vs.* 0.18% ± 0.05%). Especially for the BA mixture, the recorded length increase exceeded 0.1% by far. This normally indicates a serious sensitivity to ASR. Surprisingly, this criterion was also not met for the reference limestone concrete. Note that the length change of the BA concrete is also characterized by a much larger standard deviation on the individual values. It demonstrates that the ASR reactivity of the BA depends on its specific composition and its often high heterogeneity.

The observed expansion during the modified Oberholster test for the BA concrete is rather in contrast with literature which normally reports an acceptable ASR resistance. Perhaps the accelerated testing conditions and the fact that the exposure solution is NaOH, which also reacts with the metallic Al present in the BA, may explain this unexpected behavior. To see whether the experiment indeed resulted in expansion and cracking due to ASR, additional microscopic thin section analysis on the test samples from the Oberholster test is needed. It would also be useful to perform an additional Oberholster test on concrete with BA that was subjected to the reactive washing operation. That investigation is, for the moment, still ongoing.

#### 4.4.3. Resistance to Acid Attack

**Figure 7:**
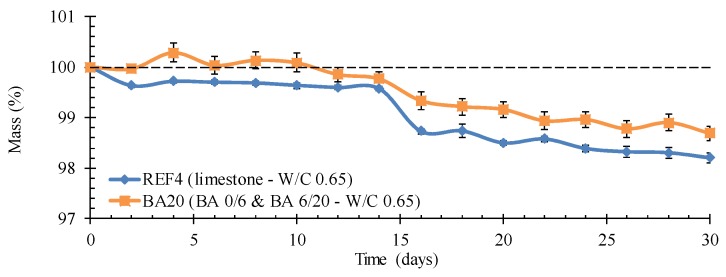
Lower mass loss of concrete BA20 *vs.* limestone concrete REF4 during cyclic exposure to a mixture of lactic and acetic acid.

**Figure 8:**
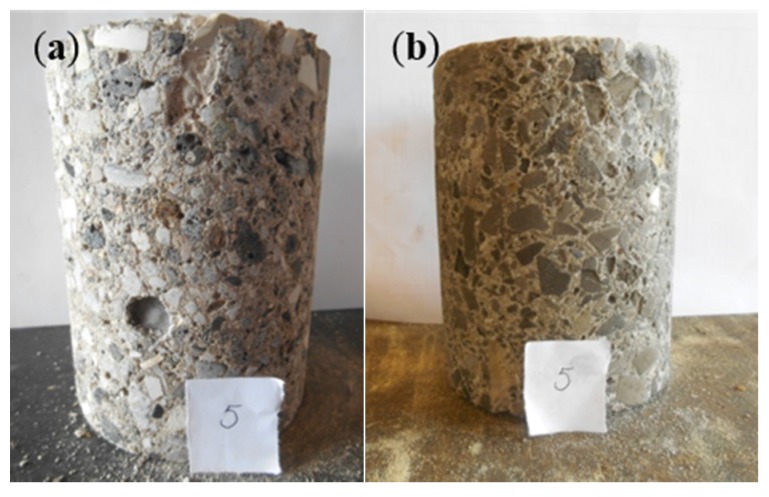
Higher superficial deterioration of concrete BA20 (**a**) *vs.* limestone concrete REF4 (**b**) after cyclic exposure to lactic and acetic acid.

From the recorded relative mass changes, concrete BA20 seems to be more resistant to cyclic lactic and acetic acid exposure than the reference limestone concrete REF4 (Figure 7). However, a visual inspection of the samples shows that one should state this conclusion rather carefully. Apparently, the reference concrete eroded rather homogenously and the sample surface remained smooth. The BA concrete showed a quite different behavior. There, only the cement matrix eroded in between the BA aggregates. This resulted in a rough sample surface and some loss of individual, mainly spherical and smooth-shaped BA grains (Figure 8). As the mass loss of individual aggregates can vary considerably per test sample, it is no surprise that the relative mass change of the BA concrete is characterized by a larger standard deviation on the individual values than the reference concrete (Figure 7).

The difference in sample deterioration between the two concrete types can be explained as follows. The lactic and acetic acid react with both the calcium hydroxide present in the cement matrix and with the limestone aggregates which are, in essence, CaCO_3_. In both cases, the reaction products are CO_2_ and H_2_O as well as either calcium acetate or lactate, depending on the acid. Thus, in the case of the reference concrete, both the cement matrix and the aggregate fraction deteriorated. It explains the still rather smooth appearance of the concrete surface after the acid exposure. As the calcium content of the BA-based aggregates is much less (around 10%–15%), it evidently contains much less of the vulnerable CaCO_3_ in comparison with the limestone aggregates of the reference concrete. In other words, just the cement matrix of the BA concrete is susceptible to the acid attack. As this cement matrix is also more porous due to the presence of more longitudinal expansion voids (Section 4.3), it is also more accessible for the acidic exposure solution. As a consequence, it will deteriorate more than the cement matrix of the reference concrete. This, in contrast with the BA-based aggregates remaining more or less intact, resulted in a rather inhomogeneous deterioration of the BA concrete. The corresponding very distinctive roughening of the concrete surface is probably as undesirable as the higher mass loss of the reference concrete.

#### 4.4.4. Resistance to Freeze-Thaw Attack

No problems were observed regarding the freeze-thaw resistance of the BA concrete. This conclusion follows from both the visual inspection and the ultrasonic testing. Visually, no damage phenomena such as cracks were observed. Secondly, the ultrasonic pulse velocity before freeze-thaw exposure (3951 ± 93 m/s) is more or less equal to the one after 14 freeze-thaw cycles (3994 ± 88 m/s). The ultrasonic pulse velocities recorded for the reference mixture did not change either (before: 4369 ± 82 m/s, after: 4320 ± 160 m/s). The difference in ultrasonic pulse velocity between the BA mixture and the reference mixture is due to the higher porosity and lower strength of the former concrete type. Note that the influence of de-icing salts on the freeze-thaw resistance was not investigated.

### 4.5. Optimization of the Bottom Ash Characteristics

#### 4.5.1. Optimization regarding Workability and Water Absorption

Initially, it seemed that the large amount of fines was causing the high water absorption of the BA. Therefore, its water absorption with exclusion of the fraction <2 mm was determined and compared to the value of the non-sieved BA. Surprisingly, it was found that the water absorption was not really affected by this sieving operation (Table 8). Also, washing the BA with tap water instead of with industrial process water (containing more fine particles) did not result in a less absorptive behavior. Although the sieving operation influenced the concrete workability in a positive manner (Table 4), this is not attributable to the omission of the fine fraction.

**Table 8:** The limited influence of excluding the fraction <2 mm and washing with tap water instead of process water on the water absorption of the fine BA.

BA Treatment	BA 0/6 Sieved and Aged	BA 2/6 Sieved and Aged	BA 0/6 Sieved and Washed (Process Water)	BA 0/6 Sieved and Washed (Tap Water)
${\mathrm{WA}}_{24}$ (%)	10.3 ± 1.6	11.8 ± 0.1	11.2 ± 0.3	11.0 ± 0.2

The high water absorption was believed to be caused by the high porosity of the BA rather than by the amount of fines. Attempts were made to separate the porous from the non-porous elements. However, this turned out rather difficult. The porous elements could also not be detected visually. For instance, quite some metal parts within the BA looked porous because they were covered by with porous-looking layers due to the sintering process during incineration. Floating tests based on the difference in density between the non-porous and porous elements were also unsuccessful. However, it could be demonstrated that crushing the aggregates in a laboratory ball mill had a positive effect on the water absorption. As can be seen in Table 9, this crushing operation mainly influenced the smaller fractions of the bottom ash (BA 0/6 (≥2 mm) and BA 6/20 (<4 mm)). In view of concrete manufacturing, it is thus advantageous to use the crushed material because the weaker porous elements are crushed. The slightly higher water absorption induced by having a somewhat greater fine fraction this way appears not to be comparable to the diminished absorption that can be obtained by removing the porous parts.

**Table 9:** The pronounced influence of a crushing operation on the reduction in water absorption of BA 0/6 (≥2 mm) and BA 6/20 (<4 mm) *vs.* BA 6/20 (≥4 mm).

BA Fraction	BA 0/6 (≥2 mm)		BA 6/20 (≥4 mm)		BA 6/20 (<4 mm)	
Crushing	Before	After	Before	After	Before	After
${\mathrm{WA}}_{24}$ (%)	9.5 ± 0.9	6.2 ± 0.9	3.4 ± 0.2	3.0 ± 0.0	12.4 ± 1.1	7.1 ± 0.4

Also pre-wetting of the BA had a distinct positive influence. However, a lot depends on the method applied. The first technique described in Section 3.9.1 did not give satisfying results. Putting the BA in contact with water to achieve a 60% saturation resulted in a too-wet, non-surface-dry aggregate. The mixtures made with this material (Table 4: mixtures BA10–BA12) showed a good workability without SP addition. Still, mixture BA10 with the highest W/C ratio (=0.65) was on the verge of segregating. The overdose of water also affected the compressive strength in a negative way (Table 5). Its strength class was only C16/20, which is lower than the required C20/25.

The second and third pre-wetting methods were more promising in terms of gaining a sufficient workability without losing too much strength performance (Table 4, Table 5: mixtures BA21–BA22). It is recommended to pre-wet the BA in advance (third method) rather than to add the same surplus water content simultaneously with the mixing water (second method). The pre-wetting establishes a water-cement film around the grains in advance which prevents further absorption by the BA aggregates. As such, the aggregates do not get saturated, a higher effective water/cement ratio is reached and the workability is less of an issue. Thus, the sequence of adding the different components during concrete manufacturing is important. After testing several amounts of water, 1 m% of the coarse BA 6/20 and 2.5 m% of the fine BA 0/6 appeared to be the optimal pre-wetting content of water. This more or less corresponds with the amount of water absorbed in the first 2 min after the addition of the mixing water. Thus, it is not necessary to consider the whole 6 min mixing timeframe.

#### 4.5.2. Optimization regarding the Susceptibility to Expansion

**Figure 9:**
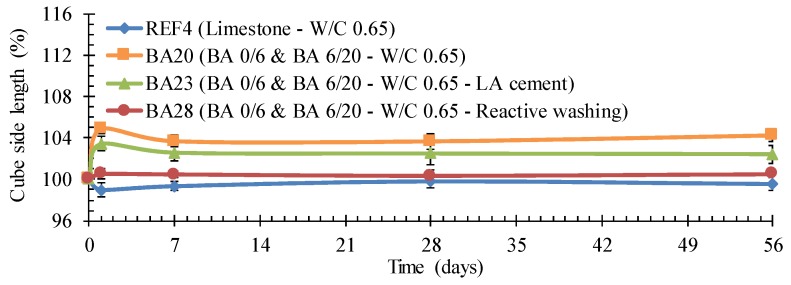
The achievable lower relative expansion of BA concrete with LA cement as binder or after reactive washing of the BA aggregates.

To diminish the expansion, LA cement could be used. From the length change measurements performed on mixture BA23, it could be concluded that the expansion was reduced from 3%–4% to 2.5%, but it was not completely eliminated (Figure 9: mixture BA23).

On the other hand, a reactive washing of the aggregates with 1 M NaOH was very effective and eliminated the problem completely (Figure 9: Mixture BA28). As shown in Table 10, reactive washing resulted in a significant decrease in Al reactivity after 14 days, especially in case of the fine BA. After only 4 h, the change in Al reactivity for both BA fractions was still non-significant.

**Table 10:** The pronounced effectiveness of a reactive washing operation on the Al reactivity of the fine and coarse BA, especially after 14 days.

Time	Al Reactivity (%)	
	BA 0/6	BA 6/20
Before reactive washing	2.22	0.16
After 4 h	1.93	0.21
After 14 days	0.73	0.02

Almost no expansion was recorded for concrete BA28 containing BA 0/6 and 6/20 washed with NaOH (Figure 9). There was a concern regarding the strength of this concrete because the surface showed signs of peeling. However, this appeared to be not an issue (Table 5: C25/30). Although this reactive washing can solve the expansion problem, one should remain aware of the fact that this chemical processing will increase the production cost of prefabricated Lego bricks significantly. Moreover, there will be some additional environmental issues. Further research is needed to see whether recycled NaOH obtained from other industrial processes can be used for this purpose.

### 4.6. Evaluation of the Lego Bricks

#### 4.6.1. Applied Concrete Mixtures

Lego brick 1 was cast using a concrete with the same composition as mixture BA20. Lego brick 2 must be seen as the reference. Its composition was the same as mixtures REF 1 and REF 4. Finally, an optimized BA concrete composition was used for the production of Lego brick 3. It contained crushed BA 0/6 (with somewhat better workability properties) instead of the sieved and washed BA 0/6. For BA 6/20, there was no difference in water absorption between the sieved and washed and the crushed aggregates, so the former was used again. Both the BA 0/6 and 6/20 were sieved afterwards to exclude the fractions smaller than 1 mm and 4 mm, respectively. This enabled a better approximation of the particle size distribution of the reference limestone 2/6 and 6/20 aggregates. Since the BA 0/6 was the most reactive due to its higher metallic Al content, this fine aggregate was subjected to the reactive washing with 1 M NaOH for two to three weeks. Prior to concrete mixing, the aggregates were pre-wetted with 2.9 l water per 180 L of concrete (1 m% of BA 6/20 and 2.5 m% of BA 0/6). A LA cement CEM I 52.5 N HES LA HSR was used as binder. The predefined W/C ratio amounted to 0.65. The compositions of all Lego bricks were included in Table 4.

#### 4.6.2. Observations Made in Fresh and Hardened State

The unadjusted BA concrete mixture (*cf.* BA20) that was used for Lego brick 1 was characterized by a poor workability. Even the addition of 10 mL SP per kg cement could not overcome the problem. This behavior can be explained by the fact that another delivery of coarse BA was used for this concrete which was characterized by a higher water absorption. Nevertheless, with some extra compaction efforts, it was possible to cast the Lego brick. All protrusions were filled in a proper manner and no difficulties were encountered during demolding. However, in hardened state, surface laitance, clear traces of the vibration needle, longitudinal expansion voids and compaction voids could be seen. The expansion itself was comparable to what was observed earlier for mixture BA20 on small concrete cubes. Despite the larger dimensions of the Lego brick, the expansion was not excessive. This can be explained by the fact that the ratio of the troweled surface over the total concrete surface available (troweled and cast surfaces) was lower for the Lego brick than for the cubes with a 150 mm side. The mechanical performance of the concrete used for Lego brick 1 was inadequate since its strength class was only C16/20. This was mainly due to the difficult compaction of the poorly workable concrete which resulted in more voids.

None of these issues were encountered when casting reference Lego brick 2. The only problem was that the concrete was too workable. Without SP addition the concrete was almost segregating. Soon after casting, a distinct water layer appeared on the surface and bleeding occurred. Apparently, applying a W/C ratio of 0.65 for a large concrete volume was too much. This is in contrast with the smaller batches of this concrete that were produced earlier on (REF1 and REF4).

**Figure 10:**
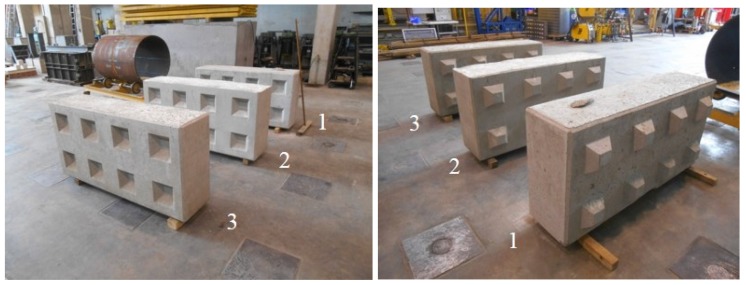
The three Lego bricks (3: the optimized BA-based Lego brick; 2: the reference limestone-based Lego brick; and 1: the BA-based unadjusted Lego brick).

Once Lego brick 3 was produced, the influence of the different optimization techniques could be evaluated. The use of crushed BA 2/6 and pre-wetting the aggregates solved the workability problem. No SP was needed anymore. The reactive washing of the fine BA 2/6 again appeared to be very effective in eliminating expansion and the longitudinal voids. The use of LA cement probably also helped to achieve this. The sieving operations performed on the BA to eliminate the excess in fine material and to approximate the particle size distributions of the traditional limestone aggregates resulted in a more evenly distributed use of sand and BA in accordance with the Fuller approach. This contributed to the better strength (C20/25) and workability (Table 4: slump: S4, flow: F5) performance as well. Figure 10 shows pictures of all three Lego bricks.

## 5. Conclusions

It is possible to produce a proper concrete in which both the fine and coarse aggregates have been replaced with BA resulting from MSWI. The concrete seems suitable for casting prefabricated Lego bricks. The minimum required strength class (=C20/25) can be assured even with W/C ratios of no less than 0.65 and without using an expensive SP.

However, a dedicated processing of the BA prior to concrete manufacturing is imperative. Excluding the excess of fines to approximate the particle size distributions of the traditional limestone aggregates is advised. This results in a better packing and a higher strength of the concrete. A crushing operation before the sieving eliminates porous elements in the BA which absorb a lot of water. As such, a better workability of the concrete can be obtained. A controlled pre-wetting of the BA just before concrete mixing in relation to the time-dependent water absorption curve also helps to improve the concrete workability. Given these techniques, it will probably be possible to manufacture a sufficiently workable BA concrete with a design W/C ratio of only 0.60 in the future.

The significant presence of metallic Al in the BA makes the concrete susceptible to the formation of longitudinal voids and expansion. The use of a LA cement can somewhat reduce this problem. A reactive washing of the BA with 1 M NaOH eliminates the problem completely. Still, the latter procedure is not ideal from both an economic and an environmental point of view. Further research on more appropriate reactive washing techniques is still needed.

No problems regarding leaching and freeze-thaw were observed for the BA concrete. On the other hand, the concrete shows a pronounced surface roughening after acetic and lactic acid exposure and does not pass the modified Oberholster test for concrete. The expansion (>0.1%) after the latter test indicates ASR sensitivity. However, further microscopic analysis is needed to see whether ASR is indeed causing the problem. Moreover, the tested BA concrete was not fully optimized yet (e.g., with exclusion of the reactive washing stage). Additional tests on the finally optimized mixture used for Lego brick 3 are, for the moment, still ongoing.
